# Application of FIB-SEM Techniques for the Advanced Characterization of Earth and Planetary Materials

**DOI:** 10.1155/2020/8406917

**Published:** 2020-07-25

**Authors:** Lixin Gu, Nian Wang, Xu Tang, H. G. Changela

**Affiliations:** ^1^Electron Microscopy Laboratory, Institute of Geology and Geophysics, Chinese Academy of Sciences, Beijing, China; ^2^Key Laboratory of Earth and Planetary Physics, Institute of Geology and Geophysics, Chinese Academy of Sciences, Beijing, China; ^3^Innovation Academy for Earth Science, Chinese Academy of Sciences, Beijing 10029, China; ^4^University of Chinese Academy of Sciences, Beijing, China; ^5^Qian Xuesen Laboratory of Space Technology, Chinese Academy of Space Technology, Beijing, China; ^6^Department of Earth & Planetary Science, University of New Mexico, New Mexico, USA

## Abstract

Advanced microanalytical techniques such as high-resolution transmission electron microscopy (HRTEM), atom probe tomography (APT), and synchrotron-based scanning transmission X-ray microscopy (STXM) enable one to characterize the structure and chemical and isotopic compositions of natural materials down towards the atomic scale. Dual focused ion beam-scanning electron microscopy (FIB-SEM) is a powerful tool for site-specific sample preparation and subsequent analysis by TEM, APT, and STXM to the highest energy and spatial resolutions. FIB-SEM also works as a stand-alone technique for three-dimensional (3D) tomography. In this review, we will outline the principles and challenges when using FIB-SEM for the advanced characterization of natural materials in the Earth and Planetary Sciences. More specifically, we aim to highlight the state-of-the-art applications of FIB-SEM using examples including (a) traditional FIB ultrathin sample preparation of small particles in the study of space weathering of lunar soil grains, (b) migration of Pb isotopes in zircons by FIB-based APT, (c) coordinated synchrotron-based STXM characterization of extraterrestrial organic material in carbonaceous chondrite, and finally (d) FIB-based 3D tomography of oil shale pores by slice and view methods. Dual beam FIB-SEM is a powerful analytical platform, the scope of which, for technological development and adaptation, is vast and exciting in the field of Earth and Planetary Sciences. For example, dual beam FIB-SEM will be a vital technique for the characterization of fine-grained asteroid and lunar samples returned to the Earth in the near future.

## 1. Introduction

Most physical, chemical, and biological processes on Earth involve the interaction of naturally occurring materials at the macroscopic, submicron to nanoscopic scale. Beyond the Earth, unique astrophysical processes are recorded in planetary materials by, for instance, high pressure impact-related minerals [1] and high temperature condensates from the early solar nebula to super nova remnants [2]. Minerals, in their naturally occurring settings, record their evolutionary histories. They determine the rock properties, displaying variation in structure and chemical compositions. Understanding them via structural, chemical, and isotopic analysis is essential for unraveling the natural history of geological and planetary materials.

In recent decades, advances in microbeam analytical technology, such as micro-Raman spectroscopy, scanning electron microscopy (SEM), transmission electron microscopy (TEM), atom-probe tomography (APT), and scanning transmission X-ray microscopy (STXM), enable one to accurately determine the morphology, crystal structure, and elemental, organic functional chemical and isotopic compositions of natural and synthetic materials at the micro-, nano-, or even the atomic scale [3–5]. However, sample preparation has long been a major obstacle that has limited the natural scientists' use of advanced techniques at the highest resolution. For instance, high-resolution TEM (HRTEM) requires the thicknesses of samples at electron transparent whilst preserving the nature of the sample. Thus, a TEM sample or “foil” should be less than 100 nm and preferably less than 50 nm for associated techniques such as electron energy loss spectroscopy (EELS) for high-quality data [6, 7]. A challenge lies in preparing such ultrathin foils with uniform thicknesses at the site of interest for geological samples, because of different sample properties, e.g., sample heterogeneity and porosity. This situation has been improved with the emergence of site-specific TEM sample preparation techniques such as the focused ion beam system.

The concept of the focused ion beam technique using a liquid Ga ion source was originally developed by Seliger and Fleming in 1974 [8, 9]. One of the successful applications of the early FIB system was used for chip failure analysis in the semiconductor industry [8]. Later, the dual beam system was built by combining the advantages of ion beam milling and high-resolution SEM imaging. The preparation of TEM foils by FIB started in the late 1980s and early 1990s. This dual-beam concept benefits from site-specific selection and controlled microscopic sample manipulation. Currently, the dual beam FIB-SEM system has become a versatile and powerful imaging and micromachining platform. Its application has been extended from the semiconductor industry to other fields such as material, life, and Earth and planetary sciences [6, 10–14].

Since then, FIB-SEM has become a diverse multifunctional tool for revealing structural, elemental, and isotopic chemical information in natural materials down to the nanometer scale. However, for their effective characterization, analytical methods require adaptation when using the dual beam FIB-SEM system. This review addresses the advanced use of FIB-SEM for a range of application in the natural sciences: from TEM analysis of small lunar soil grains, APT analysis of Pb isotopes in Earth-based zircons, and 3D slice and view of Earth-based shale kerogen to the characterization of extraterrestrial organics by coordinated synchrotron-based STXM-TEM. In addition, we address the key adaptations required in the FIB-SEM analytical methodologies for these different applications in Earth and Planetary Sciences. Finally, we assess the future development of the dual beam FIB-SEM technique.

## 2. A Brief Overview of the Dual Beam FIB-SEM System

**Figure 1:**
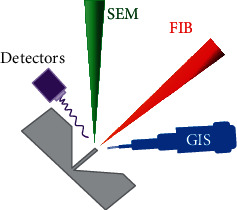
Schematic illustration of the FIB-SEM system. GIS: gas injection system.

A schematic of the FIB-SEM system is illustrated in Figure 1. The ion beam column is fixed at an angle of 52°~55° with a vertical axis of the electron beam. As for the ion source, Ga is an excellent choice due to its low melting point (29.8°C), low volatility, and low vapor pressure and being easily distinguished from other elements [15, 16]. By applying an electric field, the liquid metal is drawn into a conical shape with a diameter of 2~5 nm by the balance between electrostatic and surface tension forces [16]. Once the Ga^+^ beam is extracted from the ion gun, it is accelerated with the voltage of 1~30 kV and then passes through the condenser and objective lenses. When the incident beam scans the sample surface, various ion beam-material interactions occur, the products of which can be collected and analyzed by multiple detectors.

The interaction between Ga^+^ ions and the target material can offer imaging, milling, and deposition. These are the generic processes performed in the operation of the dual FIB-SEM system. Specific adaptations to the FIB sample preparation method however need to be tailored for the preparation of samples specific to particular analytical techniques (Section 3).

### 2.1. Imaging

**Figure 2:**
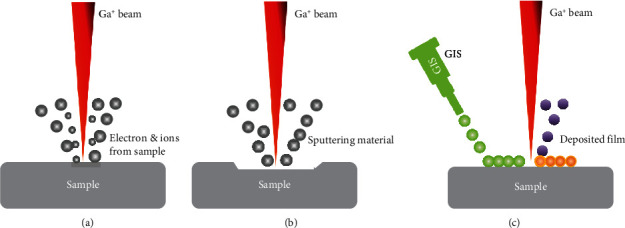
Principle of FIB (a) imaging, (b) milling, and (c) deposition (modified from [21]).

Similar to a typical SEM, the incident Ga^+^ beam can produce high-resolution secondary electron (SE) images with enhanced contrast of a sample surface, as illustrated in Figure 2(a). Due to the small volume of ion-solid interaction, the ion-induced secondary electron yield is strongly dependent on the crystallographic orientation of the target material. The much higher mass of a Ga^+^ ion than an electron means that much fewer Ga^+^ ions are backscattered, therefore producing more SEs than that from the electron beam with similar beam size and currents, producing a higher signal/noise ratio in the images. Additionally, secondary ions (SI) can also be collected at each point of a raster for producing ion images for chemical or isotopic analysis, examples being secondary ion mass spectroscopy (SIMS) [17–19].

### 2.2. Milling

Samples can be microscopically removed (also called sputtering) by ion-atom collisions [20]. A surface atom will be ejected if its kinetic energy transferred from an incident ion is sufficient to overcome the binding energy of the target atoms [6, 15]. The sputtering rate depends on the accelerating voltage, the substrate materials, and the angle of incidence [21]. For cross-section observation, a higher beam current is usually used for rough milling, followed by a lower current to make a fine polish. High-energy ions can cause uncontrolled damage on the sample, such as ion implantation and surface amorphization [22] (see Section 3). Additionally, the sputtering process can be made material selective through a process known as gas-assisted etching [15, 23]. For example, the FIB etch rate can be enhanced with the use of XeF_2_ for SiO_2_ [23].

### 2.3. Deposition

Unlike milling, the FIB-SEM system can offer precise, localized deposition of materials by using a gas injection system (GIS). In this process, the metal-containing organic compound is heated to a gas that flows out of a narrow tube above the sample surface. When ions or electrons scan a selected region, the metallic components of the precursor will be deposited on the surface of substrate [24]. To deposit an oxide insulator, siloxane gas and oxygen are used. The deposited material is not fully pure, because organic impurities as well as Ga^+^ ions are inevitably included [15]. Pt, C, W, Au, SiO_2_, and others can be deposited using the GIS system.

### 2.4. Additional Accessories

Energy-dispersive X-ray spectroscopy (EDS) is the most wildly used method for measuring major elements (0.1 at %) in a sample, and its elemental detection range is 4Be~92 U [25]. Unlike EDS, wavelength dispersive X-ray spectroscopy (WDS) improves the elemental detection limit and energy resolutions, allowing the detection and quantification of major and trace elements by comparing the measured X-ray intensity with that of a standard sample. Time-of-flight secondary ion mass spectrometry (TOF-SIMS) emerged in recent years with detection sensitivity down to ppm [26, 27]. This technique has been applied to investigate, for instance, the precipitation of boron in steel, mineralization of bone tissue, and soil sorption of heavy metals [28–30]. It has enormous application prospects in future Earth and planetary science research. The FIB-SEM can also be equipped with an electron backscatter diffraction (EBSD) detector which can be used to identify mineral phases and obtain crystallographic orientation information at the micron scale [31]. In addition, by integrating with a cathodoluminescence (CL) detector, FIB-SEM is capable of revealing the microstructures (growth zoning, deformation features, etc.), crystal chemistry (such as trace element distribution), and reconstructing geological processes [32].

## 3. Applications of the FIB-SEM System

### 3.1. FIB and TEM

It is well known that TEM has the capability to image the structure and chemistry of materials at the nanometer scale. Spherical aberration (Cs) correction techniques can extend TEM imaging to the sub-Angstrom scale. The high-angle annular dark field scanning transmission electron microscopy (HAADF-STEM) and electron energy loss spectroscopy with the energy resolution better than 10 meV have been used in condensed matter physics and material science [33]. For Earth and planetary sciences, TEM is mainly used to study nanoscale features of materials, including morphology, crystal structures, and elemental valence states of minerals [6].

Prior to the use of FIB-SEM, TEM foils of terrestrial rocks and extraterrestrial materials were prepared using ultramicrotome and argon ion milling. The ultramicrotome is widely used in the preparation of biological samples. Examples of its application are for the characterization of acid mine drainage sediments [34] to interplanetary dust particles [35]. This technique however has disadvantage of lacking site-specific preparation. The Ar^+^ milling method is effective for material sciences. However, one disadvantage of this technique is that artifacts may be introduced during mechanical thinning. Another disadvantage is that the milling region cannot be exactly controlled due to the broad ion beam. These problems have been addressed by FIB-SEM in the past two decades. The FIB system makes it easy to extract a microsized foil from the desired site on the sample surface. The foil is then attached to the grid and thinned down to the desired thickness (<100 nm).

The FIB and TEM techniques has been a routine analytical technique for terrestrial samples, such as ore minerals, natural diamonds, high-pressure experiment products, and microfossils [36–39]. Ciobanu et al. reviewed applications of FIB and advanced electron microscopy. The authors identified mineral species and traced the evolution of their intergrowths down to the atomic scale by preparing a sulphosalt-sulphide assemblage sample using the FIB-SEM platform [38]. There have been a range of studies: from the microstructure of feldspars to the characterization of platinum group minerals in Earth sciences [40–43]. The application of FIB and TEM techniques in terrestrial samples will be more popular in the future, such as characterization of dislocations and nanoinclusions in diamonds and identification of microfossils [44, 45].

**Figure 3:**
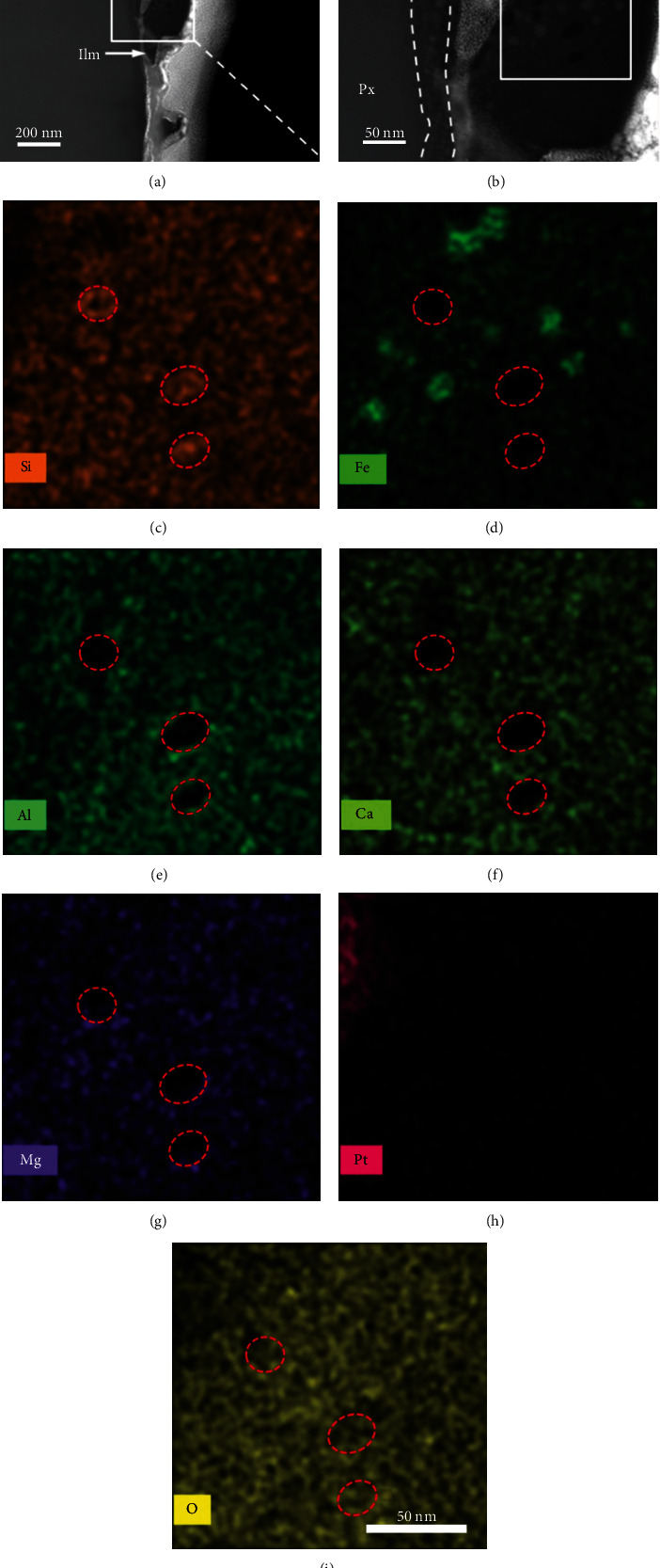
Microstructure and elemental mapping of a lunar pyroxene grain (Px) and adhering particles. (a) HAADF-STEM image of the sample. Platinum has filled between fragments and the pyroxene grain. Feldspathic fragment (Fel) and a small ilmenite (Ilm) grain were also found on the surface of pyroxene. (b) High magnification HAADF image of the Mg-Fe silicate fragment, showing the space weathered features. Dark and light particles are observed. (c–i) EDS element mapping of Si, Fe, Al, Ca, Mg, and O, suggesting that the dark particles in (b) are silicon oxide and the light particles are nanophase Fe. Reprinted with permission from Wiley and modified from [49].

The FIB-SEM is also readily used for the preparation of TEM samples in the study of extraterrestrial materials, such as presolar grains, high pressure mineral phases, and other meteorite components [10, 46–48]. An example of this is shown in Figure 3. A particular challenge lies in the analysis of the structure of small particles. In this case, FIB and TEM techniques were used to study space weathering of lunar soil grains. Space weathering is a phenomenon that occurs on the surface of Moon or other airless celestial bodies [49–51]. Previous studies have demonstrated that space weathering alters the optical properties as well as the chemical and microstructural properties of extraterrestrial bodies, by the formation of nanophase Fe (npFe) particles, vesicle structures, Fe-Si phase, and so on [52, 53]. The nanophase Fe particles are considered to cause changes in optical spectra of planetary surfaces [53]. By studying the submicron-sized adhering particles on the surface of a lunar pyroxene grain using FIB-SEM and TEM, silicon oxide nanoparticles (npSiO_x_) with npFe by-products of space weathering were observed in an Mg-Fe silicate fragment, as shown in Figure 3 [49]. The coexisting npSiO_x_ and npFe probably formed directly in micrometeorite shock-induced melt, instead of solar-wind generated vapor deposit or irradiated rim. This new observation will shed light on space weathering processes on the Moon and airless celestial bodies.

It should be noted that the high-energy ion beam will damage the sample surface, forming an amorphous layer. Previous studies have shown that accelerating voltage is a major factor affecting the thickness of amorphous layer [54, 55]. Taking silicon as an example, the thickness is ~20 nm, 2.5 nm, and 1 nm, respectively, for the 30 keV, 5 keV, and 2 keV Ga^+^ beam [54]. Surface cleaning with a lower energy Ar^+^ beam can also reduce the amorphous layer thickness. Therefore, it is necessary to design an appropriate sample preparation method according to the characteristics of different geological samples (e.g. hardness, structure, and chemical composition). For ultrathinning of submicron particles on a surface, initial E-beam capping rather than the Ga^+^ beam eliminates any possible sputtering of small particles on a surface [56]. Additionally, combining FIB with ultramicrotomy can overcome the challenges of some fine-grained samples, making them free from ion beam damage, redeposition, or curtaining [10, 57].

### 3.2. FIB and APT

**Figure 4:**
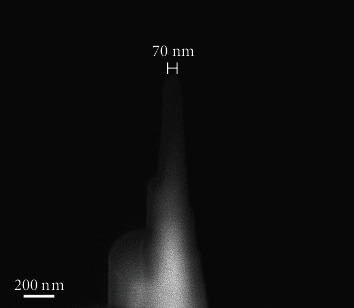
Needle-shaped specimen prepared by FIB-SEM technique.

Atom probe tomography is a kind of field ion microscopy (FIM). A pulsed voltage is applied to the needle-shaped tip sample, breaking atomic bonds at the surface. The evaporated ions are projected onto a position-sensitive detector by a strong electrostatic field. The measurement of the ion flight times, from laser pulse to detector impact, allows the ion identities to be determined by time-of-flight mass spectrometry [58]. The chemical and isotopic compositions can then be rendered in 3D at a near atomic scale [58–61]. FIB-SEM has proved vital for the microtip preparation of the sample for APT. Similar to the preparation of TEM foils, the selected site is marked, protected with deposited Pt, and then extracted with a micromanipulator [62]. After the wedge of extracted material is attached to a carrier microtip, it is then shaped into a needle-like specimen using FIB annular milling mode, as shown in Figure 4.

When applied to geological samples, which are commonly insulating, APT is usually operated in a “pulsed laser” mode that promotes field evaporation [58, 63].

**Figure 5:**
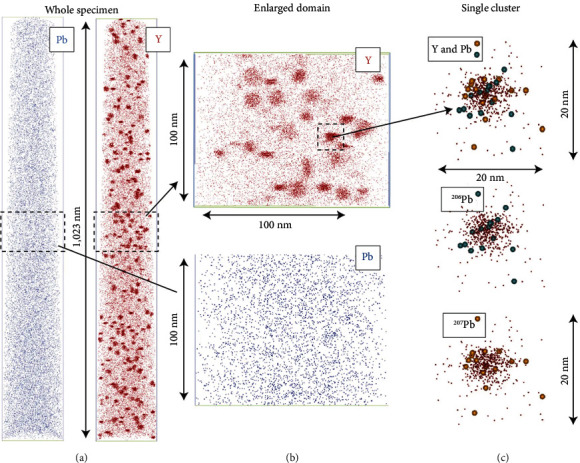
APT images of Y and Pb clusters in the 4.4-Gyr-oldzircon (reprinted with permission from Springer Nature [64]) (for interpretation of the references, the reader is referred to the web version of this article.)

Zircon is one of the best minerals for U-Pb dating due to its high physical and chemical stability. The oxygen isotopic composition of ancient zircons is useful for inferring the formation time of the hydrosphere and the habitability of the early Earth [64]. However, the reliability of ages may be biased due to an insufficient understanding of Pb migration/distribution within the zircons. Valley et al. [64] proposed FIB and APT techniques as a valuable tool for studying a 4.4-Gyr-old Hadean zircon, as shown in Figure 5. It was found that isolated nanoclusters are enriched in incompatible elements including radiogenic Pb with an unusually high ^207^Pb/^206^Pb ratio, though the average bulk value of ^207^Pb/^206^Pb is in good agreement with SIMS data at the micron scale [58, 64]. The accurate U-Pb isotopic analysis of nanoscale domains from this work opens up a new era of nanogeochronology [58, 59]. In addition, FIB and APT techniques have been also used for the study of deformation induced trace element migration and the nanoscale gold clusters in arsenopyrite [58, 65, 66].

### 3.3. FIB and Synchrotron Techniques

Synchrotron-based Scanning Transmission X-ray Microscopy (STXM) takes images of X-ray transparent samples via the raster of a stage behind a fixed highly focused X-ray beam down to the nanometer scale [67]. The use of a monochromator with energy resolution (*E*/*∆E*: ~3000) enables a stack of X-ray images to be taken at different energies around the X-ray Absorption Near Edge Fine Structure (XANES) of an element of interest. Elemental, chemical, mineralogical, and perhaps more uniquely organic functional chemical distributions can be obtained across the X-ray image [68, 69]. XANES can also be used for the determination of the oxidation state of Fe in samples [70]. Here, we focus on the use of C-K edge XANES which is now an important method of characterizing the distribution of organic functional chemical bonds in natural samples at the nanoscopic scale. This method has more recently been pertinent for the characterization of organic material (OM) in carbonaceous chondrites [69, 71] for their use as potential analogues of carbonaceous asteroid sample that will be returning to the Earth by international sample return missions in the near future [72].

**Figure 6:**
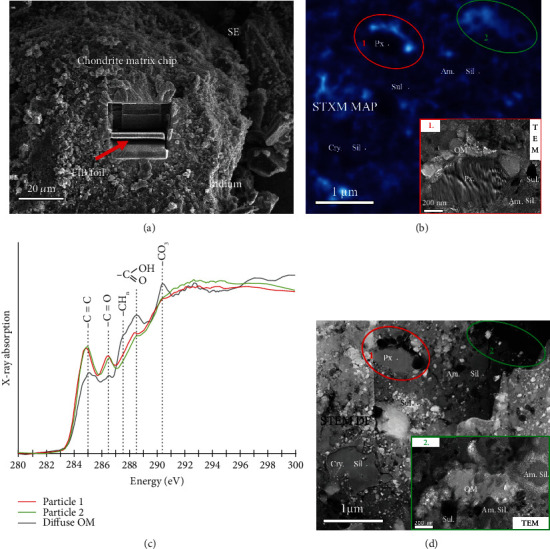
Analysis of organic matter in carbonaceous chondrites by FIB-STXM-TEM. (a) Showing the preparation of FIB foil from a chip of chondrite matrix. (b) A STXM map showing the distribution of a spectral population characteristic of aromatic-carbonyl-carboxyl/ester bearing macromolecular insoluble organic matter (bright blue features). (c) XANES spectra of organic particles 1 and 2 in (b) and diffuse spectra (fainter blue). (d) The coordinated STEM dark field image of (b) displaying the setting of the organic particles (dark grey). Note the TEM-BF images of particles 1 and 2. Particle 1 occurs as beads, and particle 2 is a vein filling the matrix. Reprinted with permission from Wiley and modified from [73].

Organic matter is found in a wide range of planetary materials such as chondrites, interplanetary dust particles (IDPs), Martian meteorites, micrometeorites, and comets. The morphology, molecular composition, and distribution of OM can better constrain its hydrothermal evolution in the early solar system [73, 74].  Figure 6 shows the example of OM in a carbonaceous chondrite studied by combined FIB-SEM, TEM, and synchrotron-based STXM technology [73]. FIB sections were extracted from the matrices of chondrites pressed in indium foil. Sample destruction is reduced by avoiding making a polished thin section and eliminating any organic contamination such as epoxy. Molecular characterization and textural features were studied by STXM and TEM, respectively. Organic matter in chondrites displays heterogeneities in XANES spectral populations. The authors discussed the evolution of chondritic OM by parent body hydrothermal processing and the possible origins of both soluble and insoluble organic components of a particular carbonaceous chondrite group. It should be noted that the C-XANES data shown here were obtained following the procedures for X-ray microscopy studies of radiation sensitive samples, which should be performed before TEM experiments in order to prevent radiation damage of the samples by the TEM electron beam [75]. The amount of radiation damage per unit of analytical information has been shown to be typically 100 times lower in STXM-based XANES spectroscopy than in TEM-based EELS [76, 77].

### 3.4. FIB for 3D Tomography

The microscopic features of rocks in 2D can be well analyzed using scanning electron microscopy. However, 3D is crucial for understanding the true distribution of phases in samples. In the dual beam system, the Ga^+^ beam can be used for serial milling of the sample, and at the same time, a sequence of SEM cross-sectional images can be obtained. Then, segmentation and 3D visualization of the sample can be rendered using commercial software (e.g., Avizo) [78]. Such 3D tomographic analysis bridges the gap between X-ray and transmission electron microscopic tomography techniques. More advanced systems are also equipped with EDS and EBSD for chemical and crystallographic data in 3D, which has recently become increasingly popular [6, 79, 80].

Taking oil shale as an example, characterization and modeling of the pores' shape and connectivity provide essential information on the permeability and gas accumulation space [81–83]. Unlike the conventional reservoirs of sandstones and carbonates, previous studies have demonstrated that the shale reservoirs are usually dominated by a large number of nanometer pores [84–86]. Hence, FIB-SEM tomography can robustly identify and characterize the microstructural properties of shale kerogen. Saif et al. characterized the heterogeneity and anisotropy of the microstructure in the Eocene Green River oil shale using X-ray computed tomography (*μ*CT), SEM, MAPS mineralogy, and FIB-SEM technology [81]. FIB-SEM 3D imaging enabled visualization and quantitative analysis of pores, organic matter, and minerals. This application provides an example for studying the heterogeneity and structure of oil shale in the laboratory. Additionally, Longmaxi shale in the southern Sichuan Basin has been used to produce industrial gas flow and is considered as a potential target region for shale gas exploration in China. Hence, the multiscale structure (organic matter and pores) of shale gas reservoirs has received extensive attention [84, 87–90]. Based on the representative volume elements selected using 2D SEM images, 3D tomography was conducted to study the pore size distribution and connectivity. Quantitative analysis and modeling provide useful information for evaluating the multiscale structure as well as the fracturing potential of strata in shale formations [87, 89].

**Figure 7:**
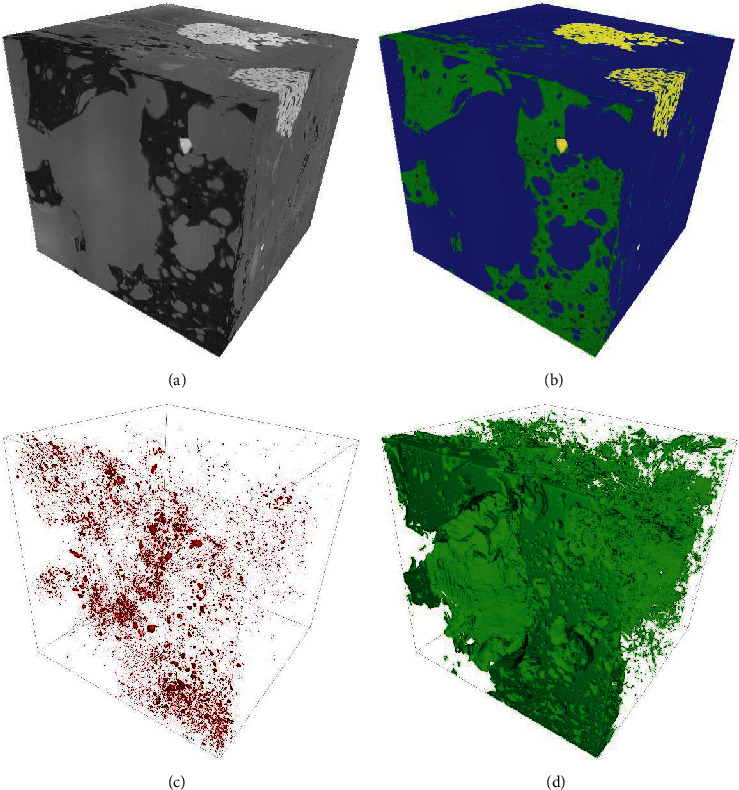
A typical 3D structure of shale sample from Hunan Province, China. (a, b) Three-phase segmentation of mineral (blue), organic matter (green), and pore (red). (c, d) The visualization of segmented pores and organic matter.

A typical 3D structure of shale is shown in Figure 7. We can clearly distinguish minerals, organic matter, and pores after the segmentation. As most geological materials have a low electrical conductivity, C or Au should be deposited to reduce charging effects before 3D tomography. The porosity and volume of organic matter were calculated as 0.36% and 11.75%, respectively, by using digital rock 3D models reconstructed from FIB-SEM tomography image dataset. Detailed analytical procedures can be found elsewhere [37, 80].

The volume of FIB 3D tomography is usually about ~1000 *μ*m^3^. Due to the limited analytical scales, 3D reconstruction results are sometimes not fully representative of the actual complex and highly heterogeneous structure of rocks. It is therefore necessary to combine X-ray computed tomography and SEM mosaic images for statistical analysis from micron to nanometer scale [91, 92]. Therefore, a balance should be found between “resolution” and “analytical scale” during the research of materials such as oil shale. In addition, shale gas reservoir evaluation also requires total organic carbon content (TOC) analysis, geochemical parameter analysis, well logging physical property analysis, and so on.

TEM for small particles, APT, STXM, and 3D tomography all have to apply the FIB technique in different ways for the successful preparation of samples and the minimization of sample damage. Ultimately, FIB-SEM is a partially destructive technique and the interaction of Ga^+^ ions on various materials can alter their properties. It is therefore vital to prepare samples using methods minimizing the alteration of the material to be characterized. This is arguably even more important for labile, soft, and low Z number materials such as organic material that are susceptible to the remobilization of their functional groups by Ga^+^ interactions. Here, we summarize variations on the conditions and give specific suggestions required for the different applications using FIB for either TEM, APT, 3D tomography, or organic-based STXM analyses (Table 1).

### 3.5. Future Trends

As discussed above, although the FIB-SEM dual-beam system has been essential and widely used on natural materials, there are still some technical limitations: (1) high-energy Ga^+^ ion beam implantation causes damage during sample preparation and (2) cross-section milling in FIB-SEM 3D tomography often has the “curtain” effect, which affects the imaging resolution and causes reconstruction artifacts [101]. Improvements in low kV Ga^+^ beam and advances in multiple beam techniques have shown great potential to address these issues. In addition, the application of the FIB-SEM system will be further extended with the development of new technologies.

### 3.6. Multiple Ion Beam Microscopy

Higher ion beam currents for improved signal/noise and higher spatial resolutions are common goals in microscopy techniques. As mentioned above, most FIB-SEM ion beam systems use a liquid Ga ion source. The ion beam current of commercial FIB system is less than 100 nA, and the best resolution is about 2.5 nm at 30 kV, as shown in Table 2. More recently, other types of ion sources such as He, Xe, and Ne have been developed as promising sources for many applications [25, 102, 103]. Helium ion microscopy has been proven to be an alternative to SEM and FIB in some fields providing higher resolution (~0.5 nm) and a larger depth of field, which can help one obtain finer details on material surfaces with no charging effects. This is an important characteristic for studying the microstructure of insulating minerals and uncoated biological samples [88, 104].

Xe^+^ plasma FIB microscopy (PFIB) has a slightly lower resolution, but its beam current is about 20 times as large as the Ga ion beam. Higher beam current means higher sputtering rates and larger analytical volumes at the same time. This PFIB system has been employed in semiconductor industry for milling materials at hundreds of micron length scale. In addition, previous studies have shown that the depth of damage caused by the Xe^+^ plasma FIB is 20~40% less than that measured from a Ga^+^ FIB-prepared specimen [102, 105]. Thus, ultrathin TEM foils with higher quality can be prepared using this technique. In the future, “resolution” and “milling efficiency” will be both improved with the development of new techniques and combination of multiple ion beams.

### 3.7. Other Complementary Techniques

Compared with using FIB-SEM as a stand-alone instrument, synergistic techniques show unique advantages that enable us to acquire more information from one sample and unravel the complex natural processes. This has been used to reveal shock-induced trace element segregation [106], microbe-mineral interactions [107], and the evolution of extraterrestrial materials [108]. However, comprehensive in situ analysis remains challenged by the different requirements of various instrument platforms and sample preparation. Motivated by the new analytical possibilities, the Parallel Ion Electron Spectrometry (PIES) instrument integrated with FIB, TEM, and SIMS in a single system was recently built by Eswara [109, 110]. This technique can offer high-resolution imaging and high-sensitivity analysis of elements, which may have many application prospects in studying the microstructure of materials and minerals.

Additionally, with the use of the sample preparation technique of FIB-SEM system, mass spectroscopy techniques such as thermal ionization mass spectrometry (TIMS) and quadrupole inductively coupled plasma mass spectrometry (Q-ICP-MS) could offer some level of spatial resolution that was not typically available [111]. In the future, FIB will remain an important link between different microanalytical platforms from the macroscopic to microscopic scale, which will further promote the development of analytical methods and new technologies for the advanced characterization of the Earth and planetary materials.

## 4. Conclusion

1. FIB-SEM is a powerful tool for site-specific sample preparation of TEM ultrathin foils and APT needle-shaped samples of natural and synthetic materials and also can work as a stand-alone technique for three-dimensional (3D) tomography. This technique has been widely used to study the microstructure, chemical and isotopic compositions of terrestrial rocks, extraterrestrial materials, paleontology, and marine sciences from the micron scale down to the single-atom resolution, through combining with multiple detectors and other in situ analytical instruments, including TEM, APT, and synchrotron-based STXM as examples
2. FIB methodologies need to be tailored for different purposes, whether it is small particle analysis, atomic probe tomography, synchrotron-based scanning transmission X-ray microscopy, or 3D FIB-SEM tomography
3. Multiple ion beam microscopy and some new technologies that have emerged recently are expected to be used to study the Earth and extraterrestrial materials for better revealing complex natural processes

## Figures and Tables

**Table 1:** FIB as an independent instrument or combined with other techniques.

Synergistic techniques	Features	Target materials	Conditions and suggestions	References
Stand-alone FIB-SEM	Cross-section imaging or 3D tomography integrated with multiple detectors	Clay minerals and oil shales (this study)	(1) Medium current to balance milling speed and minimized curtain effect (2) Pt coated for protective layer (3) Acquire high-resolution SE and BSE image simultaneously for later image segmentation	[92–94]

FIB and TEM	Microstructural and crystallographic characterization	Earth & planetary materials such as ore minerals, high pressure phases and extraterrestrial materials	(1) Stepping down the polishing currents to 10~50 pA/5 kV or even lower (2) For irregular small particles, E-beam for Pt deposition is necessary	[36, 42, 56, 95, 96]

FIB and APT	3D chemical and isotopic information	U-Th-Pb isotope systems & trace element compositions in zircon, monazite etc.	(1) Pt/Au-coated as protective layer—evaporation field of carbon is too high (2) Make a mark for selected position if the area of interest is relatively small	[64, 65, 97–99]

FIB and synchrotron techniques	Elemental mapping	Terrestrial shale kerogen organic carbon & extraterrestrial organic matter in planetary materials, e.g., carbonaceous chondrites.	(1) None carbon capping. Ideally no EXPOXY embedding (2) Low current for final milling and low KV E-beam imaging to minimize damage (3) Ensure STXM is performed prior to TEM on organic materials due to alteration of organic by high energy TEM beam	[68, 73, 100]

**Table 2:** Overview of ion beam current and resolution in commercial FIB systems.

FIB	Beam current	Resolution
Ga	0.1-100 nA	~3 nm
He	0.1-100 pA	~0.5 nm
Ne	0.1-100 pA	~1.9 nm
Xe	0.1-2 *μ*A	<20 nm
